# Screening Methods for Age-Related Hearing Loss in Older Patients with Cancer: A Review of the Literature

**DOI:** 10.3390/geriatrics3030048

**Published:** 2018-08-02

**Authors:** Michelle Lycke, Tessa Lefebvre, Lieselot Cool, Koen Van Eygen, Tom Boterberg, Patricia Schofield, Philip R. Debruyne

**Affiliations:** 1 Cancer Centre, Department of Medical Oncology, General Hospital Groeninge, B-8500 Kortrijk, Belgium; michelle.lycke@azgroeninge.be (M.L.); tessa.lefebvre@azgroeninge.be (T.L.); lieselot.cool@azgroeninge.be (L.C.); 2 Department of Radiotherapy and Experimental Cancer Research, Ghent University, B-9000 Ghent, Belgium; tom.boterberg@ugent.be; 3 Cancer Centre, Department of Haematology, General Hospital Groeninge, B-8500 Kortrijk, Belgium; koen.vaneygen@azgroeninge.be; 4 Faculty of Health, Social Care and Education, Anglia Ruskin University, Chelmsford CM1 1SQ, UK; patricia.schofield@anglia.ac.uk

**Keywords:** cancer, age-related hearing loss, screening, eHealth

## Abstract

As people grow older, they may experience loss in hearing sensitivity. Age-related hearing loss may negatively affect the patient’s quality of life as it may lead to social isolation. In older patients with cancer, hearing loss can seriously interfere with the patient’s ability to deal properly with all aspects of their disease, and may have a cumulative effect on their already decreased quality of life. Therefore, the proper screening of those conditions is essential in order to optimise the patient’s comfort during and after treatment. This review article aims at providing a concise image of the nature of age-related hearing loss, and provides an overview of the screening methods that could be used in older patients with cancer.

## 1. Introduction

Older cancer patients are a vulnerable group of individuals, as they may have developed multiple comorbidities as a consequence of ageing. One of those comorbidities is age-related hearing loss (ARHL), also referred to as presbyacusis or presbycusis. Although its onset and rate of progression may vary widely among individuals, the prevalence of ARHL increases with age. A review by Roth et al. (2005) indicated that at the age of 70, 20% of European women and 30% of European men present with a loss of at least 30 decibel Hearing Loss (dB HL). At the age of 80, numbers increase to 55% of men and 45% of women being affected [1]. In the United States of America (USA), similar numbers are reported [2]. In males, the first signs of presbyacusis may already be detected around the third to fourth decade, when the higher frequency range from 10.0 kHz to 16.0 kHz is affected. During the fourth decade, damage in lower frequencies ranging from 6.0 kHz to 8.0 kHz can be found. In females, ARHL starts later in life. The first signs in women are usually noticed a decade later than in males. Although authors remain inconclusive, some attribute these changes to the more frequently occurring, often work-related, noise exposure in men [3]. Ovarian steroid hormones and cardiovascular diseases that affect inner ear blood flow have also been reported to account for the better hearing thresholds in women [4]. In both men and women, decreased hearing sensitivity in the most important region for speech ranging from 0.5 kHz to 4.0 kHz, starts around the sixth life decade [5]. Besides these differences in gender, it is also reported that Caucasian individuals can be more affected by AHRL than their African-American counterparts [6]. 

Presbyacusis is known to have a serious impact on patients’ daily life. It not only affects their physical, cognitive, and emotional activities, it also influences patients’ social functioning. As patients lose their hearing, they feel left out as they have difficulties communicating with others. Even without hearing loss, it has been reported that listening effort increases with age as a result of age-related declines in cognitive functioning, hearing sensitivity, and peripheral auditory functions [7]. As a result, their quality of life deteriorates with various symptoms such as depression, social isolation, and lowered self-esteem [8]. Further, ARHL is also associated with a future loss of functional abilities and functional dependence [9]. However, despite its prevalence and morbidity, presbyacusis is often left unrecognised and untreated. It is reported that only 25% of the patients who have a hearing loss great enough to be aided with hearing aids actually receive proper rehabilitation [10]. On the contrary, many people who own hearing aids do not use them on a regular basis, and even when they are wearing them, some still have socially disabling levels of hearing loss [11]. There are multiple reasons why patients are somewhat reluctant towards hearing aids. The process of accepting the need of hearing aids, selecting, trying, and purchasing them, and using them afterwards is challenging for many patients. However, the most common barrier is the dissatisfaction with its performance [12]. Patients often believe that wearing hearing aids can be compared to wearing glasses. Once you put them on, you hear as you heard before, which is not the case. During the adaptation process, they often feel discouraged when the device does not exactly perform as they had hoped. Conversations in noisy environments remain difficult and are often unpleasant due to the excessive background noise. As a result, the hearing aids disappear in their closets and are almost never used [13,14].

Despite these difficulties, in cancer patients, who already have a lower quality of life, the optimisation of such a condition is crucial. Therefore, the National Comprehensive Cancer Network (NCCN) guidelines in Older Adult Oncology recommend an assessment of sensory functions such as vision and hearing [15] as part of a comprehensive geriatric assessment (CGA). The latter can be described as a multidimensional evaluation of the health of the older patient with cancer and aims at exploring vulnerabilities in various health domains in order to decrease the risk of morbidity and mortality [16]. Within a CGA, it is not the main objective to diagnose patients as having a significant hearing loss and refer them to a hearing aid specialist. The purpose is merely to select those patients who show signs of decreased hearing sensitivity so that proper measures can be taken. One of those measures can include a referral to the otolaryngologist for a full examination or referral to a hearing aid specialist. Others measures can include taking notes, a more pronounced articulation, etc. 

## 2. Screening Methods for Age-Related Hearing Loss in Older Patients with Cancer

### 2.1. Methodology

Over the years, many screening methods for age-related hearing loss have been described. Screening tests were identified by searching the electronic peer-reviewed databases MEDLINE and Web of Science. In a first search, the search strategy was kept broad and consisted only of terms for ‘hearing’ (e.g., ‘hearing’, ‘audiology’, ‘audiological’) and the term ‘screening’. In a second search, once several screening tools were selected, a more detailed searching strategy was used, as it then consisted out of the term ‘screening’ and the term of the specific screening tool (e.g., ‘whispered voice’, ‘otoacoustic emissions’). The reference list of selected manuscripts was then further checked for eligible screening tools. 

Table 1 gives an overview of all of the types of tests that were selected through the literature review that are included within this overview. While no consensus has been reached on which tool is the most optimal one to implement within the CGA, this table describes the advantages and disadvantages of each tool when used in our target population as part of a CGA. 

### 2.2. Whispered Voice Test

The NCCN Guidelines in Older Adult Oncology recommend using a quick and simple screening tool. The test is performed by whispering the sentence “What is your name?” while standing behind the patient and occluding one ear. The patient fails if he does not hear the sentence. The test is repeated for the contralateral ear [15,17]. Free-field voice testing was one of the easiest methods to get an idea of someone’s hearing, and was used as the standard method before clinical audiometers became available in the 1940s. The test proposed by the NCCN is not validated; therefore, several issues arise. Nonetheless, several researchers have published reliable forms of whispered voice testing [18,19,20]. All of the proposed tests use more or less the same methodology. The examiner has to stand behind the patient in order to remove the ability of speech-reading. In addition, the non-tested ear has to be excluded to rule out interference. The easiest way to mask the non-tested ear is to gently occlude the external auditory canal with a finger while rubbing it in a circular manner. Whispering is done after the examiner exhaled completely. This setup is similar in all types of whispered voice tests. The distance between the patient and the examiner, and the words expressed to the patient may differ. Swan et al. (1985) described a whispered voice test (WVT) to identify those individuals who may benefit from management of their hearing loss. In their test, the patient needed to repeat a combination of three numerals and letters. A new combination was expressed when the patient repeated an incorrect combination or if he or she did not repeat anything at all. The researcher did not mention the distance between the patient and the examiner [18]. Macphee et al. (1985) investigated whether the WVT could be implemented for use in a group of patients admitted to a geriatric unit. They performed the test in multiple conditions: conversational voice at six inches and at two feet from the ear, and whispered voice at six inches and at two feet from the ear. Results indicated that using a whispered voice at two feet was the most discriminant test with a sensitivity of 100% and specificity of 84% [19]. Eekhof et al. (1996) compared the WVT to other screening measures. The first was the Pat-225, which is a handheld device that produces mixed noise with frequencies ranging from 0.5 kHz to 4.0 kHz at 30 dB HL. The second was the AudioScope-3, which is an otoscope with a built-in screening audiometer that produces pure tones at 0.5 kHz, 1.0 kHz, 2.0 kHz, and 4.0 kHz. Eekhof et al. considered an inability of hearing tones at 40 dB HL as having a hearing loss of 40 dB or greater. The last instrument was a screening audiometer called the Micromate-304, which could only generate tones at 2.0 kHz and 4.0 kHz at 40 dB HL. Patients passed the test if they could hear both tones. Results indicated that the WVT was the best tool to use. Although the WVT showed promising results, they were the first to report that they had found a broad variation between the outcomes of several examiners [20]. The problem with the WVT is that the words are pronounced live and are not pre-recorded. Therefore, it is difficult to standardise the technique, control the pitch of the whisper, and control the background noise. Further, different acoustic environments may also play a role in the outcome of the test [20,21,22]. Researchers have investigated the effect of experience on the sensitivity and specificity of the WVT. They have found that the sound intensity of the whispered voice of experienced examiners is 8 dB to 10 dB higher than the whisper of an examiner without experience. They state that this problem can be addressed by training through voice measurement in order to ensure an nearly equal loud intensity of the whispers [23]. In a more recent trial by Labanca et al. (2017), the WVT was examined when using specific words or phrases in a group of older individuals. Besides the area under the curve (AUC), they have also examined the inter-rater reliability (IRR). The words and phrases with the highest AUC and IRR values were: “shoe” (AUC = 0.918; IRR = 0.877), “window” (AUC = 0.917; IRR = 0.869), “it looks like it’s going to rain” (AUC = 0.911; IRR = 0.810), and “the bus is late” (AUC = 0.900; IRR = 0.810). Although they concluded that these results demonstrate that the WVT is a useful screening tool for detecting hearing loss in older subjects, it should be noted that this trial was conducted with Portuguese-speaking individuals. Therefore, it is possible that these results cannot be extrapolated to subjects with other native languages [24]. Our research group has examined the performance of the WVT in a group of older cancer patients as part of the CGA. Results showed an excellent specificity (100.0%), but poor sensitivity (30.0%), hampering the use of this screening tool in clinical practice [25]. 

### 2.3. AudioScope

As the WVT has many problems when accounting for the IRR, researchers have examined other, more objective measures to screen for hearing loss. A popular objective tool is the AudioScope (Welch Allyn Medical Products, Skaneateles Falls, NY, USA) [26]. The AudioScope is a handheld otoscope that is able to generate a set of pure tones at 0.5 kHz, 1.0 kHz, 2.0 kHz, and 4.0 kHz with an intensity of either 25 dB HL or 40 dB HL. It also enables an inspection of the tympanic membrane and auditory canal. The examiner needs to hold the AudioScope directly into the external auditory canal. A probe tip on the device occludes the canal. The listener is asked to indicate whether the given tone is heard. For screening purposes, a pass is given when the patient hears the pure tone at 40 dB HL. Although the AudioScope has an excellent diagnostic accuracy, the device provided by Welch Allyn is rather expensive; it may cost up to €600 [10,27,28,29].

### 2.4. Screening Questionnaires

A widely used screening questionnaire concerns the Hearing Handicap Inventory for the Elderly (HHIE). Since its development in 1982 by Ventry and Weinstein, the HHIE has gained a lot of interest in terms of screening for hearing loss in an older population [30]. The HHIE is a self-assessment tool containing 25 items on emotional and situational problems that are associated with hearing loss. The HHIE helps determine the need for rehabilitation and assists in the planning and implementation of an aural rehabilitation program. Weinstein et al. (1986) reported that the test–retest reliability for the questionnaire was high for both face-to-face as well as for paper-and-pencil administration [31]. Ventry and Weinstein published a shortened version of their self-administering questionnaire, including 10 items [32]. Per question, a score with a maximum of four is given, leading to a total of 40 points. Final scores of at least 26 indicate an 84% probability of having a hearing impairment and a moderate to severe handicap [29,33]. Lichtenstein et al. (1988) examined the diagnostic performance of the HHIE screening version (HHIE-S) against different definitions of hearing loss. Sensitivity ranged from 53% to 72%, whereas specificity reached a maximum of 84%. They concluded that the HHIE-S was a robust and valid test for identifying older individuals with a hearing impairment, irrespective of the definition used to define the patient’s hearing status [34]. Over the years, the HHIE-S has attracted interest and been translated and subsequently validated in several languages [35,36,37]. The HHIE-S is easy and straightforward. In a recent trial by our group, the HHIE was examined in a group of older patients with cancer as part of a CGA. While reaching a high specificity (100.0%), sensitivity was calculated as 0.0%. This could be explained by none of the patients who had a hearing loss, as measured with conventional audiometry, scoring high enough on the HHIE [25]. However, it should be noted that the HHIE measures functional and not physical hearing loss, which may account for these poor sensitivity results [10]. 

### 2.5. Pure Tone Audiometry

Pure tone audiometry is known as the gold standard to assess hearing loss since its introduction as a clinical tool more than 70 years ago. A necessary requirement for optimal testing is a controlled test environment with ambient noise levels as close to 0 dB sound pressure level (SPL) (ISO 8253) [38]. In that way, environmental noise masking the hearing thresholds is prevented [39]. Therefore, pure tone testing is usually performed in a sound-isolated room or sound booth. Both the American Speech Hearing Association (ASHA) and the British Society of Audiology (BSA) have published similar guidelines on how to perform pure tone audiometry [40,41]. Although pure tone audiometry is considered as the gold standard for assessing a person’s hearing, it is less suited to implement within the CGA, as it requires a transfer to the audiology department, and may be cumbersome for patients who are bedridden or who have a limited mobility due to their intravenous infusion. Therefore, there is a need for an inexpensive bedside screening tool that enables health care providers to make a quick judgement of the patient’s hearing.

In our developing world, eHealth applications are gaining more and more interest. Using information and communication in healthcare can result in improved healthcare access and improved quality of service delivery [42]. Swanepoel et al. (2010) described several eHealth applications in audiology in a systematic review [43]. However, the most interesting tools are those developed for use on mobile phones running on Android, iOS, or other operating systems (OS), since mobile phones are small and can easily be carried around. Swanepoel et al. (2014) developed the HearScreen™ (HearX Group, Pretoria, South Africa) application on an inexpensive Android OS cell phone. The application was validated in a group of school children. The idea behind the screening is somewhat similar to that of the AudioScope. The children hear a specific frequency (1.0 kHz, 2.0 kHz, or 4.0 kHz) at an intensity level of 25 dB HL. A pass or fail score is given. HearScreen™ showed similar results to those of screening with conventional audiometry [44]. It should be mentioned that while the Android OS cell phone may be inexpensive, the Hearscreen™ app has a purchase cost of $600 [45]. Wenjin et al. (2014) evaluated the Smart Hearing app in school children. Smart Hearing runs on Android OS, and is able to deliver pure tones (1.0 kHz, 2.0 kHz, and 4.0 kHz) at an intensity level ranging from 20 dB HL to 60 dB HL. The pass or fail screening cut-off was set at >30 dB HL. Results indicated a low sensitivity of 37.5% and a high specificity of 92.6%. They concluded that further improvements of the app needed to be undertaken in order to improve the sensitivity of the test [46]. Na et al. (2014) developed a screening tool that runs on Android software. Although their test is not yet available for public use, it showed some promising results. The researchers used the built-in microphone of the smartphone to measure the surrounding environmental noise. As a result, the known threshold shift was much lower than it is in other mobile hearing screening apps [47]. Other apps, such as Hearing Test for Android users, have also been described. However, no accuracy results have been published [48]

EarTrumpet, AudCal, and uHear™ all run on iOS devices such as an iPod, iPad, or iPhone. EarTrumpet (PraxisBiosciences, Irvine, CA, USA) was released in the iTunes Store in 2010, and is calibrated with standard Apple earbuds. The test is performed according to the Hughson–Westlake technique and allows basic (0.5 kHz, 1.0 kHz, 4.0 kHz and 8.0 kHz), comprehensive (0.25–8.0 kHz), or custom testing. Test tones are pulsating with an 0.8-s duration while the silence interval varies between one and two seconds. Masking is done automatically when a difference of at least 35 dB is found between both ears. In a quiet room, 94% (95% confidence interval (CI): 87–100%) of the thresholds detected with EarTrumpet were within 10 dB of the pure tone threshold obtained with conventional audiometry [49]. Derin et al. (2016) confirmed that EarTrumpet gives reliable results [50]. AudCal was developed by Larrosa et al. (2015) and was designed as a pure tone air conduction screening tool to aid in the calculation of a hearing handicap. Although the authors found promising results, they did not calibrate the tool. Further, the AudCal app has a less user-friendly interface, and does not offer automatic audiometric testing [51]. Kam et al. (2012) developed a computerised self-administered hearing test that can be used on iOS devices. An iPhone 3GS with iOS4 software and standard iPhone earbuds were used. Although the application seemed promising, it is not freely available in the iTunes Store [52]. uHear™ on the other hand, is a freely available iOS-based application that has been more frequently examined by several independent researchers in the field. uHear™ was developed by Unitron (Kitchener, ON, Canada) by Don Hayes, the director of the audiology department. It offers three tests: (1) a hearing sensitivity test that takes approximately five minutes to administer; (2) a one-minute speech-in-noise test; and (3) a 12-item questionnaire in order to create a hearing profile. Of all the available tests, the hearing sensitivity test is most examined. Pure tone air conduction thresholds at 0.25 kHz, 0.5 kHz, 1.0 kHz, 2.0 kHz, 4.0 kHz, and 6.0 kHz are established. The results are presented in a graphic display [53,54,55,56]. Szudek et al. (2012) were the first to report on the diagnostic performance of the tool. They evaluated 100 adult subjects and compared the results of the uHear™ app to conventional audiometry based on the pure tone average (PTA). With a sensitivity of 98% and a specificity of 82%, they concluded that uHear™ could be used as a screening tool to rule out moderate hearing loss [53]. Khoza-Shangase et al. (2013) determined the accuracy of uHear™ in young children with a mean age of nine years old. They stated that the screening tool was not as accurate as conventional audiometry in determining pure tone thresholds in school-aged children. They attributed the differences between both tests to the ambient noise levels that were present during uHear™ testing and the tool’s lack of calibration [54]. Peer et al. (2015) tested 50 ears in three environments: a waiting room, a quiet room, and a soundproof room. Sensitivity was excellent (100%) in all of the conditions. Specificity differed, and was 88% in the soundproof room, 73% in the quiet room, and 68% in the waiting room [55]. The most recently published trial on the performance of uHear™ was conducted by Abu-Ghanem et al. (2016) on older patients. They included 26 subjects with a mean age of 84.4 ± 6.7 years, and detected a sensitivity of 100% and specificity of 60% compared to conventional audiometry. They also evaluated the hearing questionnaire included in the app; however, results were less accurate than those of the hearing sensitivity test [56]. Our group has also conducted research with the uHear application in older patients with cancer. To our knowledge, we were the first to explore the use of an eHealth application in this population as part of the CGA. Although uHear showed promising results, it can be disputed whether uHear™ is the best screening tool to implement within the CGA. It has without doubt some benefits, such as its accessibility, its user-friendly interface, its short administration time, and its diagnostic accuracy, but the ongoing need for improvement cannot be ignored, as it showed a large discrepancy between threshold levels measured by the tool and standard pure tone audiometry [25,57].

### 2.6. Otoacoustic Emissions

Another measure is otoacoustic emissions (OAEs). OAEs are an objective and fast measure to establish the function of the outer hair cells within the cochlea. They are widely used as a neonatal screening method to establish an audiological diagnosis. When conducting the test, brief acoustic stimuli are produced into the external ear canal. As a result, OAEs can be measured, which are low-volume sounds that originate from the cochlea [58]. OAEs can be divided into transient-evoked (TEOAE) and distortion-product (DPOAE) OAEs, based on the type of stimuli that is presented to the patient. TEOAEs are measured within the 0.5 kHz to 4.0 kHz frequency range, and use a brief pure tone stimulus, while DPOAEs include frequencies up to 8.0 kHz, and use two pure tones that are produced simultaneously [59,60]. Although it is known that the amplitude of OAEs decreases with increasing age and increasing hearing thresholds, OAEs are not optimal to use within the CGA, as the test is highly influenced by environmental noise [61,62]. 

### 2.7. Speech-In-Noise Test

Another method to define the patient’s hearing is by use of a speech-in-noise (SPIN) test, as this type of test gives more accurate results in terms of speech intelligibility. SPIN tests give an idea of the patient’s ability to understand meaningful sentences in noisy situations, and provide valuable information, as listeners find themselves surrounded by background noise multiple times a day. The ability to understand speech-in-noise is usually presented as the speech reception threshold, which can be defined as the signal-to-noise ratio that an individual requires in order to understand 50% of the presented speech material [63]. As standard SPIN testing also requires a transfer to the audiology department, researchers have developed SPIN screening versions. One of those variants is the digit triplet test. In the digit triplet test, a series of digit triplets is presented to the subject along with background noise at a fixed or adaptive intensity, depending on the method used. Smits et al. (2004) were the first to examine the use of a Dutch SPIN test as a screening tool at home by use of a telephone in a general population. The results indicated a high sensitivity (91%) as well as a high specificity (93%) [63]. A recent trial by Vercammen et al. (2017) reported that a Flemish version of the digit triplet test, which was used with broadband noise, had a high sensitivity and high specificity to detect high-frequency hearing impairment in middle-aged persons [64]. Although SPIN tests are commonly used to assess patients’ speech intelligibility, there are only a few eHealth applications available that include SPIN tests. As previously mentioned, uHear™ offers a one-minute SPIN test. However, it has not been used in clinical trials. Potgieter et al. (2015) developed a smartphone-based digit-in-noise test to use in English-speaking South African individuals. They concluded that the mean speech reception threshold and speech recognition functions of their newly develop app corresponded to those of previously developed telephone-based digits-in-noise tests [65]. While more research is necessary for this type of screening, an eHealth SPIN screening test could be more beneficial for use in our target population, as well as in our target setting. SPIN tests have the benefit of being suprathreshold, and are therefore less influenced by environmental use compared with other screening tests based on pure tones or OAEs. They further can be conducted with inexpensive software, as no absolute calibration is necessary. Next, when using a digit triplet SPIN test, the test can be administered quickly [66]. However, one main problem with this type of test is that it is language-specific, and there are only a few languages for which eHealth SPIN tests are available. Further, no research has been conducted examining the use of SPIN tests in older patients with cancer as part of a CGA. Therefore, SPIN tests cannot be proposed as a routine screening method within the CGA at this point [66,67]. 

## 3. Conclusions

At present, an optimal screening tool to assess hearing loss in older patients with cancer is lacking. As a result, most geriatric oncology teams do not implement a hearing screening when assessing the CGA. However, the hearing assessment as part of the CGA should not be included to define the patient’s total vulnerability. It should be used as an important informative assessment, as knowing if the patient has a reduced hearing is crucial for cancer patients who need to hear and understand a lot of information on their diagnosis, its treatment, and possible treatment-related side effects. Older patients are very proud of their independence, and in many cases, sensory dysfunctions including vision and hearing loss are one of the first functional declines that they have to deal with. Although wearing glasses is well accepted socially by both young and older people, hearing aids are not that well accepted by many persons. As a result, older patients tend to hide a hearing loss, which, in an onco-geriatric population, may lead to missing essential information about the cancer diagnosis, its treatment, and/or its treatment-related adverse events [68]. If healthcare providers know that the patient has trouble hearing, they can easily adapt their speech, improve the signal-to-noise ratio, make sure they have a good pronunciation, give written instructions, make sure the patient has heard the information, etc. Providing a hearing screening as part of the CGA is the first step towards addressing hearing loss in the older patient with cancer in an objective manner. 

Until a validated tool for use within the CGA is available, it is advised to incorporate a subjective assessment of the patient’s hearing as a screening method. If the person conducting the CGA feels that the patient has a hearing loss, this should be mentioned in the CGA report. Ideally, a pop-up regarding this matter should appear when any healthcare provider opens the patient’s medical record. It is not advised to ask the patient about his or her beliefs of their hearing, as our results indicated a large discrepancy between patient-reported hearing loss and objective audiometry results. Objective screening should at this point include pure tone audiometry. Future trials should focus on the development of an eHealth tool providing a SPIN test. Preferably, this SPIN test should be kept short, as listening effort is often increased in older subjects, and assessment of such a longer test could enhance fatigue, which is already present in many cancer patients. Nonetheless, this type of test will provide healthcare professionals with an adequate overview of how the patient experiences listening in daily situations. 

## Figures and Tables

**Table 1:** Advantages and disadvantages of the selected screening methods to assess hearing loss in older patients with cancer.

Type of Test	Screening Method	Ease of Application	Influenced by Background Noise	Test–Retest Reliability	Performance	Cost
**Whispered voice testing**	Subjective	Quick Easy All published versions use more or less the same methodology Not all versions of the whispered voice test have been validated Words are pronounced live	Yes	Low Test results may be influenced by the experience level of the examiner Difficult to standardise the technique	Good diagnostic performance	Low
**AudioScope**	Objective	Quick Easy Allows inspection of the tympanic membrane and auditory canal	Yes	High	Less data available from clinical trials Excellent diagnostic accuracy	High
**Screening questionnaires (e.g., Hearing Handicap Inventory for the Elderly)**	Subjective	Self-assessment tool Short version available Validated in several languages	No	High	Individuals may overestimate or underestimate their actual hearing loss Robust and valid test for identifying older persons with a hearing impairment Excellent specificity, but poor sensitivity in older patients with cancer	Low
**Pure tone audiometry**						
- Standard	Objective	Assessment in a sound booth Requires a transfer to the audiology department	No	High test	Gold standard for detecting hearing loss	High
- eHealth	Objective	Allows bedside screening Several versions available for mobile phones running on Android and iOS software Some applications use calibrated headphones/ear buds, whereas others don’t	Yes	No information	Large variations in diagnostic accuracy	Low
**Otoacoustic emissions**	Objective	Automatic test Fast Used as screening method in neonates	Yes	High	Good diagnostic accuracy	High
**Speech-in-noise testing**						
- Standard	Objective	Requires a transfer to the audiology department No absolute calibration necessary	No	High	Good diagnostic performance	Low
- Digit triplet test		Screening version Quick Allows screening by telephone Language-specific No validated eHealth version available in many languages No absolute calibration necessary	No	High	Excellent diagnostic performance	Low
